# Aquaporins: More Than Functional Monomers in a Tetrameric Arrangement

**DOI:** 10.3390/cells7110209

**Published:** 2018-11-11

**Authors:** Marcelo Ozu, Luciano Galizia, Cynthia Acuña, Gabriela Amodeo

**Affiliations:** 1 Departamento de Biodiversidad y Biología Experimental, Facultad de Ciencias Exactas y Naturales, Universidad de Buenos Aires, Buenos Aires C1428EGA, Argentina; mozu@bg.fcen.uba.ar (M.O.); cin.flo.acu@gmail.com (C.A.); 2 Instituto de Biodiversidad y Biología Experimental y Aplicada (IBBEA), Universidad de Buenos Aires y Consejo Nacional de Investigaciones Científicas y Técnicas (CONICET), Buenos Aires C1428EGA CABA, Argentina; 3 Instituto de investigaciones Médicas A. Lanari, Facultad de Medicina, Universidad de Buenos Aires, Buenos Aires C1427ARO, Argentina; lgalizia@gmail.com; 4 Consejo Nacional de Investigaciones Científicas y Técnicas, Laboratorio de Canales Iónicos, Instituto de Investigaciones Médicas (IDIM), Universidad de Buenos Aires, Buenos Aires C1427ARO, Argentina

**Keywords:** water channel, osmotic permeability, gating, cooperative mechanism

## Abstract

Aquaporins (AQPs) function as tetrameric structures in which each monomer has its own permeable pathway. The combination of structural biology, molecular dynamics simulations, and experimental approaches has contributed to improve our knowledge of how protein conformational changes can challenge its transport capacity, rapidly altering the membrane permeability. This review is focused on evidence that highlights the functional relationship between the monomers and the tetramer. In this sense, we address AQP permeation capacity as well as regulatory mechanisms that affect the monomer, the tetramer, or tetramers combined in complex structures. We therefore explore: (i) water permeation and recent evidence on ion permeation, including the permeation pathway controversy—each monomer versus the central pore of the tetramer—and (ii) regulatory mechanisms that cannot be attributed to independent monomers. In particular, we discuss channel gating and AQPs that sense membrane tension. For the latter we propose a possible mechanism that includes the monomer (slight changes of pore shape, the number of possible H-bonds between water molecules and pore-lining residues) and the tetramer (interactions among monomers and a positive cooperative effect).

## 1. Introduction

One of the milestones in the study of water movement through biological membranes was unraveling the molecular entity, i.e., the identification of the aqueous pore or specific water channel—later named aquaporin, AQP—that could increase the membrane water permeability (P*_f_*) beyond the values expected for a simple partition-diffusion process [1,2]. The requirement of aqueous pores to hold up high water permeability values—reported in specific cells or epithelia—has been largely discussed and proposed in the biophysical community for more than sixty years [3,4,5]. The biophysical arguments introduced by Finkelstein and others sustained that if water moves by a partition-diffusion process during osmotic events, the ratio between the osmotic (P*_f_*) and diffusion (P*_d_*) permeability should be 1 (P*_f_*/P*_d_* = 1), while a ratio higher than 1 should indicate that water could be moving through pores [5,6]. The overexpression of a red cell channel-intrinsic protein (CHIP28, now AQP1) in Xenopus oocytes, unambiguously confirmed that oocyte plasma membrane can dramatically increase P*_f_* [1].

The measurement of P*_f_* immediately spread as a convenient tool among other parameters to test the impact of aquaporins in the water transfer capacity of a certain membrane. In the last two decades, structural studies have made AQPs one of the best structurally characterized membrane protein families, providing detailed information regarding the substrate specificity, mechanism of conduction and modes of regulation [7,8].

**Figure 1:**
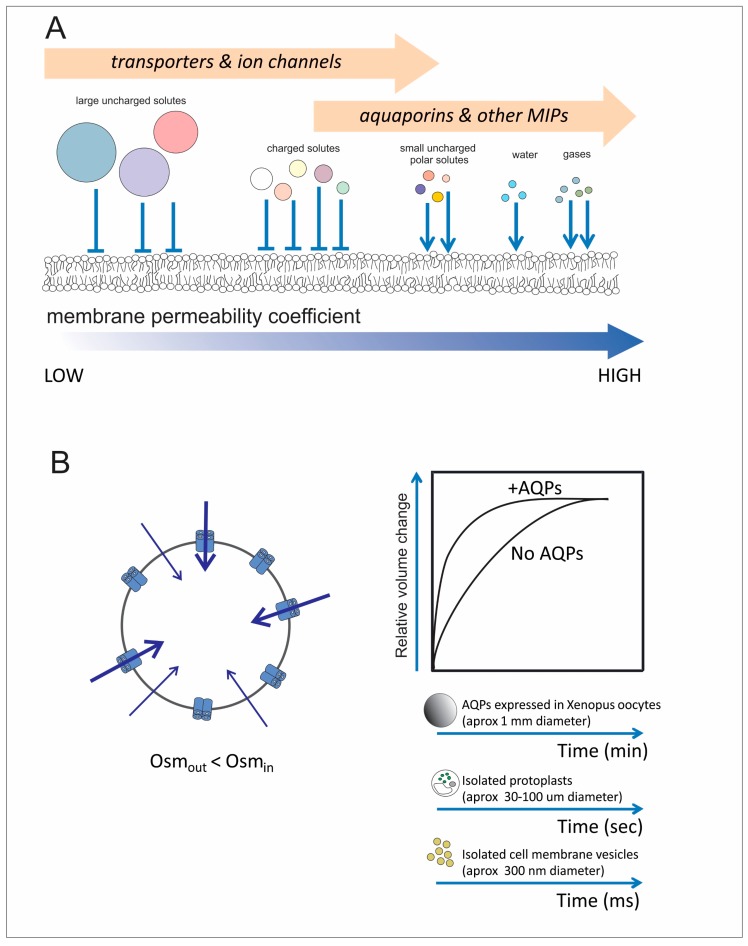
Biological membranes and aquaporins. (**A**) Biological membranes are selectively permeable. Large solutes and polar ones (e.g., ions) have very low permeability coefficients and request specific protein transporters to facilitate their transfer. On the other hand, small uncharged polar solutes as well as water and gases have less resistance to permeate through the phospholipid bilayer and protein transporters are optional. However, now we know specific channels (aquaporins) are crucial molecular entities for controlling/regulating the rate of exchange of water, gases and certain solutes including ions in certain cases. In the scheme, the arrows represent the capacity to increase the membrane permeability by introducing integral membrane proteins into the phospholipid bilayer. We propose there is an overlap in the type of transporters that can be responsible for regulating the permeation pathway of a specific solute/water/gas. (**B**) Water exchange is facilitated when AQPs that are water channels are present. As the phospholipid bilayer is also permeable to water, osmotic swelling is possible under an imposed osmotic gradient even in the absence of aquaporins. However, their presence allows a faster swelling response. In the cartoon it is represented the water entry and the consequent cell swelling imposed by the osmotic gradient. Different techniques are available to measure water membrane permeability in isolated cells or smaller structures.

In terms of permeation, AQPs belong to a widespread and complex superfamily of intrinsic membrane proteins that include AQPs that are highly permeable to water (known as orthodox aquaporins) and AQPs which might differ in their water transport capacity but facilitate the passage of small solutes (non-orthodox AQPs, including aquaglyceroporins, metalloido-porins and others) [9,10,11,12] (Figure 1A). Also, the transport of some gases is being reported in AQPs from different kingdoms [13,14], and the ion transport is being reported in certain AQPs [15,16]. A group of aquaporins with highly deviated Asparagine-Proline-Alanine (NPA) motifs was named as S-aquaporins (superfamily, small basic intrinsic protein (SIP)-like and subcellular-aquaporins) and should be considered in this classification [17].

**Figure 2:**
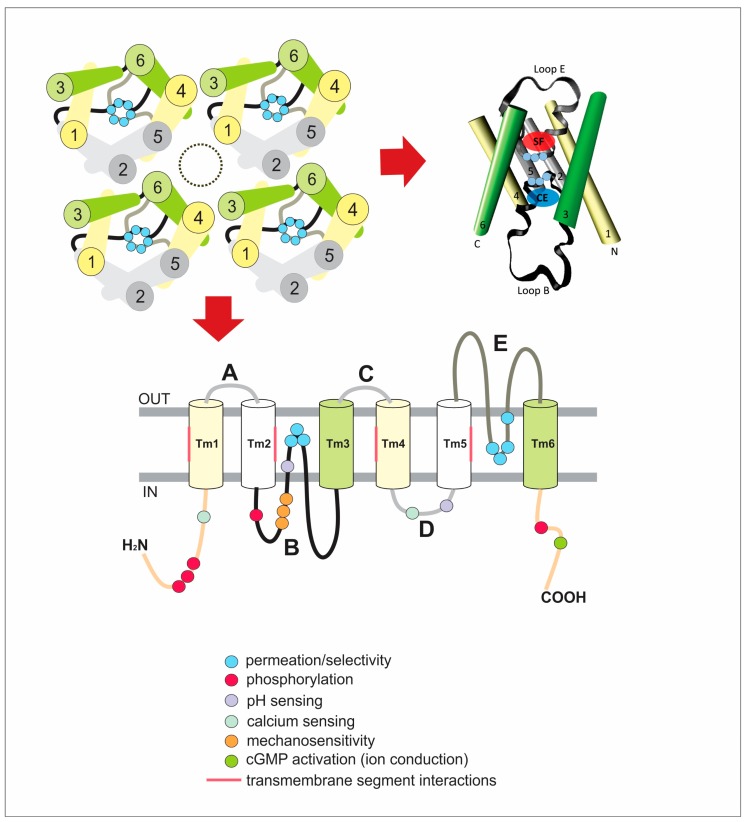
Scheme of the general structure of AQPs. (**A**) Illustration of the tetrameric organization of AQPs. Transmembrane segments (TM) are numbered from 1 to 6. The cytoplasmic loop B (black line), the extracellular loop E (gray line) and the highly conserved NPA motifs (light blue circles) are represented. The central pore is indicated by the dash-lined oval. (**B**) Lateral view of one monomer. The structure representation was created with the program Visual Molecular Dynamics (VMD) (http://www.ks.uiuc.edu/Research/vmd/) [18] using the structural data of AQP1 (pdb 1FQY) [19]. Color code and TM numbering are the same as in A. N and C represent both cytoplasmic N- and C-terminal ends. The approximated location of the selectivity filter (SF) and the cytoplasmic entrance (CE) to the single-file region are shown by transparent red and blue ovals, respectively. For detailed description see the text. (**C**) Schematic diagram of one monomer exhibiting its six transmembrane domains and loops. Again, the highly conserved NPA motifs and Ar/R selectivity filter are indicated in light blue circles. The topology includes information obtained from different aquaporins with the purpose of highlighting the residues or motifs that are (might be) critical for the described regulatory mechanisms. For a detailed description see the text.

In terms of their quaternary structure, all AQPs are tetramers of four pores, i.e., each monomer constitutes a functionally independent pore, which is quite different from the nature of the pore of classical ion channels (Figure 2). However, the tetrameric assembly creates also a central (extra) channel of mostly hydrophobic nature with no clear role/function that it is still matter of discussion (gas/ion permeation?) [11].

In terms of conduction, an overwhelming amount of evidence (site-directed mutagenesis, structural determinations, molecular dynamics simulations, etc.) has contributed to deep inside the mechanism. Studies performed in AQP1 transformed this orthodox AQP in a channel able to transport urea, glycerol, ammonia, and even protons by manipulating the diameter of the pore by means of point mutations in the selectivity filter [20]. First high-resolution AQP structures allowed hypothesizing how water molecules move in single file through the channel with an exclusion mechanism preventing proton conduction by a Grotthuss mechanism [21,22]. Deeping inside these mechanisms continues to introduce unexpected developments in the subject [8].

In terms of regulation, one of the first and most confirmed regulatory mechanism described for mammalian AQPs was trafficking, whereby AQP membrane abundance and, consequently, membrane water permeability is regulated by their relocalization in intracellular storage sites [23,24]. Among a series of papers that predicted this regulatory mode to control membrane P*_f_* years before AQPs were discovered are those that describe the nature of the water permeability adjustments induced by antidiuretic hormone in renal epithelia [25]. In this work the authors wrote: “We find that even in the unstimulated bladder, water probably crosses the luminal membrane primarily through small aqueous pores, and that this is almost certainly the case after antidiuretic hormone (ADH) stimulation. I suggest that ADH stimulation ultimately leads either to formation (or enlargement) of pores, by the rearrangement of preexisting subunits, or to an unplugging of these pores”. Freeze fracture studies confirmed the appearance of intra-membrane particle aggregates in the ADH target membranes; the number was related to the change in water permeability [26]. These observations remarkably emphasize not only the impact of water channels to sustain the P*_f_* but also their hormonal-controlled trafficking as a highly regulated mechanism with a physiological role. Overwhelming evidence has confirmed the role of AQP2 and other AQPs in renal physiology and pathophysiology [27,28]. Hormonal or environmental signals can alter P*_f_* through this mechanism in a wide variety of eukaryotic aquaporins [29,30,31].

Provided the information available from structural studies, it becomes clear that certain reoccurring structural features are responsible for how aquaporin regulation might occur. At the level of the monomer, conformational changes can reversible control water flux by means of opening and closing the pore. This was nicely confirmed for pH, phosphorylation status and calcium [32,33,34]. The word gating was taken from the ion channel field to describe the capacity to modulate the membrane P*_f_*. However, it must be emphasized that in most of ion channels, the control of transmembrane flux of ions is driven by large conformational changes that affect the pore-forming subunits. As in the case of trafficking, AQP gating provides a fast-response mechanism to control water fluxes in biological membranes to rapidly adjust P*_f_*.

Orthodox AQPs have proven to be one of the fastest reported transporters, probably sustained by their sophisticated but precise structural design. There is no straightforward answer to understand why four separate water channels should come together. Is it solely a request to warranty an impact in water flux capacity? Is it a way of providing a cooperative effect in quaternary structure to extend its regulatory properties? Although first evidence showed that lack of cooperativity between monomers was possible [35] different experimental approaches confirmed there are examples of cooperativity [36,37,38,39]. Certain aquaporins have proven to employ more complex arrangements: heterotetramerization, by means of combining oligomeric structures from isoforms o other AQPs. This has extensively been covered in studies performed in animal AQP4 [40], AQP0 [41] and plant Protoplast Intrinsic Proteins (PIPs) [42]. To what extent do gating and oligomeric rearrangements interact?

In this review, we explore evidence that highlights the functional relationship between the monomers and the tetramer. We attempt to integrate the current knowledge of the monomeric structural features that impact on the membrane water permeability in the context of a tetrameric structure. Within this frame, we review well-known regulatory mechanisms that rapidly adjust the unitary channel permeability (pH, phosphorylation, calcium, heterotetramerization), discuss the role of the tetramer on the ion transport capacity and the chain of events involved in a possible mechanism for the regulation mediated by membrane tension changes. This approach seeks to expand the current outlook of the structure-function relationship of aquaporins.

## 2. AQPs and Their Impact in Swelling Kinetics

### 2.1. Membrane Water Permeability

The initial proposal of considering AQPs as continuously open pores was challenged by evidence that certain aquaporins might remain in the membrane but modify their contribution to the osmotic water permeability (P*_f_* in upper case) by means of a gating mechanism. A high P*_f_* will allow fast volume changes in a time scale that is dependent on cell volume, i.e., in the scale of msec for isolated membrane vesicles/liposomes (circa 10^−5^ cm diameter); seconds for isolated cells (circa 10^−3^ cm diameter) and minutes for (*Xenopus*) oocytes (circa 10^−1^ cm diameter) (Figure 1B). This analysis is considered for those AQPs that are highly involved in water transfers, i.e., usually referred as the orthodox ones.

One of the first and best studied aquaporins is AQP1 (originally named CHIP28, [1]). When AQP1 is expressed in Xenopus oocytes, membrane water permeability increases up to 20-fold above water-injected oocytes for a given osmotic gradient (P*_f_* = 0.02 cm·s^−1^, compared to P*_f_* = 0.001 cm·s^−1^). However, in this system it is not always straight forward to calculate the unitary—also indicated as monomeric or channel unit—water permeability (p*_f_*, in lower case) due to uncertainty in the level of the protein expression. Thus, the calculation of the unitary water permeability might introduce errors since two possible expressions can be used:

p*_f_* = P*_f_* × area/n_monomers_ (1)

p*_f_* = P*_f_* × area/n_tetramers_ (2)

where n_monomers_ and n_tetramers_ are the number of monomers and tetramers of AQP, respectively.

Studies performed with closed lipid vesicles containing two-dimensional crystallization of AQP1 facilitated these calculations, as they provided higher accuracy in evaluating protein density [43]. As described in this study, these crystalline vesicles showed a P*_f_* = 0.472 cm·s^−1^ and allowed predicting a unitary osmotic permeability coefficient (p*_f_*) of ~5.4 × 10^−14^ cm^3^·s^−1^. The findings were also consistent with measurements performed for AQP1 in different systems showing p*_f_* values between 6–16 × 10^−14^ cm^3^·s^−1^ [44,45,46,47].

How is this translated to cell-swelling kinetics? AQP1 is a 28 kDa-protein surprisingly abundant in human red cells (~1,400,000 copies per erythrocyte) [48]. In red cells, AQP1 is therefore considered to be a main water pathway as the membrane can express other AQP types but with much lower P*_f_* values.

Based on the calculated unit water permeability of AQP1, the AQP1-mediated osmotic permeability (P*_f_*) of a human red cell can be estimated as:

P*_f_* = (p*_f_* of AQP1 × number of AQP1 per red cell)/(surface area of red cell) ~0.017 cm·s^−1^ [44]

In the literature, P*_f_* values for intact red cells were reported to be as high as 0.02 cm·s^−1^ [5,49]. If we consider the single-file model (water molecules can pass through the pore in a single-file mode) the rate turns to 1.8 × 10^9^ water molecules per monomer per second. This value is much higher than those reported to move ions in channels (10^6^ ions per second) or transporters (10^−2^–10^−4^ per second).

High P*_f_* values can be found in more complex structures, as an epithelium where cells are polarized. As an example, in hepatic epithelial cells expressing AQP8 strong differences between apical (canalicular) and basolateral P*_f_* have been described [50]. Canalicular membrane vesicles could achieve very high P*_f_* values (0.066 ± 0.014 cm·s^−1^) and these values can dramatically change if stimulation is avoided [50]. Trans-epithelial net volume-flux measurements in collecting duct epithelial cells expressing AQP2 suggested an osmotic flow rectification dependent on an asymmetrical modulation of P*_f_* between apical and basolateral membranes [51].

These observations can be extended to other kingdoms. AQP1 in red cells is abundant but represents 3.5% of membrane proteins. In the plant kingdom, aquaporins constitute 5–20% of all integral membrane proteins and although reported P*_f_* varies, selective-water channels as PIPs (mainly expressed at the level of the plasma membrane) are consistently showing high P*_f_* [52]. In addition, the number of PIP homologs is large, and evidence shows that heterotetramerization between different PIPs can also modify intrinsic water permeability [53].

Nevertheless, the abovementioned calculations fit with the hypothesis that specialized cells, or ordinary cells submitted to specific challenges, can employ PIPs and other AQPs to regulate the rate of swelling (fast or slow) by adjusting the number of inserted proteins in the membrane and/or opening and closing the channel, provided that: (i) the requested density of membrane protein is feasible, (ii) reported P*_f_* values obtained from the characterization of certain AQPs in Xenopus oocytes are consistently high, and (iii) short term regulatory properties are well described to provide a mechanism to rapidly modify P*_f_*.

### 2.2. Molecular Basis of Water Permeation

In single-file transport, the diffusion of water is governed by the capacity of water molecules to form H-bonds [54]. Molecular dynamics simulations predict that the water passage through the single-file region is limited by the time required for breaking H-bonds, reorientation of water molecules and forming new H-bonds [54]. This capacity depends on the number of pore-lining residues capable of forming H-bonds and the effective space for water molecules to form such H-bonds. Therefore, water transport through AQPs is considered not frictionless [55].

When entering the single-file region of orthodox AQPs, each water molecule loses two of the four H-bonds formed with other water molecules. Only the H-bonds with the preceding and the following are maintained allowing water molecules in the single-file lumen to form H-bonds with the pore-lining residues [55].

Molecular dynamics simulations performed with structural data obtained at 0.88 Å resolution predicted that the translocation of water molecules through the water pathway of the yeast aquaporin AQY1 occurs in a highly correlated manner at both the selectivity filter and the cytoplasmic half of the channel [22]. The selectivity filter of AQY1 has six residues capable of forming H-Bonds. However, due to steric hindrance only a fraction of the available sites is simultaneously occupied [22].

Inside the pore of rat AQP4 there are eight water molecules in the single-file region. The surface of the channel is hydrophobic, except for hydrophilic regions formed by the oxygen atoms of the main-chain carbonyl groups of H95, G94, G93 (loop B), G209, A210 and S211 (loop E) and nitrogen atoms of the side-chains amide groups N97 and N213 of the NPA motifs [56]. The electrostatic field created by the short α-helices of loops B and E forces the water molecules to orient their oxygen to the amide groups of N97 and N213 allowing H-bonds with the NPA motifs. In this region the pore is 3 Å of diameter, and with the exceptions of the two N the other residues lining the constriction are hydrophobic. Except for the central water molecule, all the rest can form H-bonds with upper and lower neighbors and one more with any of the pore-lining residues. Thus, the arrangement of the carbonyl and amide groups in the water pathway of AQP4 allows water molecules to form H-bonds with both N residues, which lowers the energy barrier to enter the constriction and allows for very fast water permeation [56].

Recent results show that the number of H-bonds between water molecules and pore-lining residues determines the permeability of the monomeric (or unitary) water pathway (p*_f_*) [54]. In addition, the charge effect at the pore entrance can account for small p*_f_* changes, constituting a fine tuning over the hundred-fold change of p*_f_* that the number of H-bond forming pore-lining residues can account for [55]. While negative charges at the pore entrance or exit have no effect on p*_f_*, positive charges increase p*_f_*, possibly because positively charged residues are weakly hydrated in comparison to negatively charged residues [55].

In the cytoplasmic half of human AQP4, H95 (loop B) can move and form H-bond with C178 (loop D), constituting a possible regulatory mechanism for water permeability [57]. The displacement of H95 is triggered by intracellular proton concentration, determining the open state at alkaline pH and the close state at acidic pH [58]. This mechanism, predicted by molecular dynamics (MD) simulations, was thought to be similar to the well-described pH regulation of SoPIP2;1 [8,59,60]. However, this hypothesis was dismissed because the proximity of H95 to a conserved glutamic acid (E41) would be responsible for the stabilization of the closed state due to the trapping of the positive charge of H95 in the electrostatic field generated by E41 [58].

## 3. Structural Changes That Regulate Permeation

### 3.1. Regulation of the Water Transport Capacity of the Monomer by pH, Phosphorylaton and Calcium

Different kind of stimuli/factors such as pH changes, calcium concentration and phosphorylation have nicely been demonstrated to regulate P*_f_* of certain AQPs. Although each stimulus elicits changes of different nature on a specific AQP, a common feature is the existence of reversible conformational changes of the pore structure that affects the water permeation through the monomeric pathway. Since this subject has been extensively reviewed [8,31,59,60,61], we just mention here the most relevant aspects. For example, pH, calcium, and phosphorylation have been shown to regulate water osmotic permeability on a wide range of AQPs in different kingdoms.

In plant PIPs the mechanism involves a displacement of a flexible loop external to the channel pore [33,62]. This gating mechanism was proposed based on the high-resolution crystal structure of spinach SoPIP2;1—in closed and open conformation—and in combination with molecular dynamics studies [33,60,63]. In the closed state the cytosolic loop D interacts with the N-terminal, producing the insertion of a hydrophobic residue (L197) into the monomer. This closed state is thought to be stabilized in the presence of Ca^2+^ by interactions with key residues of the loop D (H193), loop B (S115) and the N-terminal [33,59]. When S115 is dephosphorylated, it also stabilizes the closed state; however, phosphorylation of this residue and other key serine residues at the loop D and C-terminal disrupts this stabilization and might induce an open state [33]. Also, when the pH-sensitive residue at the loop D (H193) is protonated, the closed state is induced. This kind of mechanism involving loop movement which caps the monomer water pathway was also proposed for *Pichia pastoris* AQY1 in response to phosphorylation [59]. In AQY1, the closure mechanism might involve a unique conformation of the cytosolic N-terminus, which thereby closes the channel [64].

In contrast, another proposed mechanism of gating for other AQPs (e.g., AQP0, AQPZ, AQP4) implies smaller movements of few residues or a single one which pinch in upon the Ar/R constriction region and reduce the monomer size [59,60]. For example, in AQP0, pH and calcium regulation of P*_f_* was reported. External histidine residues located in loop A (H40) and C (H122) of the bovine AQP0 could play a role in pH sensing [65]. Based on crystal structures [66,67], a pinching mechanism of pH gating was proposed for AQP0 [59], in which small conformational changes due to protonation pinch the Ar/R region where a unique tyrosine residue (Y23) is critical in limiting the passage of water molecules. Recent MD simulations confirms the role of this tyrosine residue in the pore occlusion by pH [68]. Regarding calcium regulation of AQP0, Ca^2+^-calmodulin interaction effect on AQP0 gating was also demonstrated. Molecular dynamics simulations and functional studies of bovine AQP0 revealed the role of Ca^2+^–CaM binding on P*_f_* reduction suggesting the role of another unique tyrosine residue (Y149) located at the pore [37], producing an allosteric regulation. Recently the same group studied the molecular mechanism of this allosteric regulation of P*_f_*, as they were able to identify an unexplored region, an arginine-rich loop that connects fourth and fifth transmembrane helices, adjacent to Y149 that is proposed to be crucial in the gating of AQP0 [69].

In the case of *E. coli* AQPZ, MD simulations suggested spontaneous displacements of the well conserved arginine of the Ar/R selective filter R189 [70] and structural studies confirmed this proposed gating mechanism [71].

Regarding the role of Histidines, MD simulations predict that the homologous H81 of the plant aquaporin AtTIP2;1 moves spontaneously reaching positions that correlate with water permeation events that differ up to one order of magnitude [72]. While H95 is responsible for pH gating in human AQP4 [58], H81 is not responsible for pH gating in AtPIP2;1. Located in loop D, H131 was identified as the key residue in the pH regulation of AtPIP2;1 [73]. Besides this difference, both H95 in human AQP4 and H81 in AtTIP2;1 are in loop B, very close to the first NPA and flanked by highly conserved Glycine residues that are H-bond donors for water molecules. In the other half side of the channel, the conserved Histidine of the selectivity filter is highly mobile.

From this evidence, two different schematic pictures describe the gating of transmembrane water channels: “capping” (plant PIPs) and “pinching” (AQP) [60]. Although phosphorylation has been deeply described as being part of trafficking mechanisms in almost all mammal AQPs [74,75,76,77], it also seems to be an intrinsic step of the gating process in certain AQPs, such as AQP4 [78] and FaPIP2;1 [79].

### 3.2. AQPs That also Permeate Ions: The Central Pore versus Monomer-Permeation Controversy

The controversial issue on ion permeation through aquaporins demands special attention because the transport of ions through channels requires a strict control to maintain cellular homeostasis. Then, if aquaporins allow ion permeation, mechanisms eliciting reversible structural modifications are needed to allow and control ion translocation. To sustain transmembrane pH and ion gradients that are critical in cell homeostasis, proton and cation permeation should be strictly regulated [19]. In this section, we will introduce the discussion on the mechanisms for ion permeation through AQPs.

From the beginning, it has been well known that protons are incapable of being translocated through AQP1 [44]. The mechanism of proton exclusion was predicted to be directed by an electrostatic barrier centered on the fingerprint NPA motif, and not by the interruption of the hydrogen-bonded water chain [80]. Further experimental studies using AQP1-mutants expanded this conception, enhancing the idea that the NPA and the Ar/R constriction regions are two concerted cation filters [20,81]. In particular, the Asparagine residues of the NPA region are postulated to repel positive charges by electrostatic interactions [82,83]. Since the electrostatic profile of AQP monomers is key in selectivity [84], then, the translocation of positive charged ions through AQP1 monomers should be restricted [83]. Can the electrostatic field that repels cations attract anions? More than a decade ago it was suggested that the NPA region functions as an anion trap, preventing anions from further conduction in GlpF [85]. However, some recent evidence confirmed the permeation of anions through monomers [83,86]. Here we will focus in three aquaporins that have been investigated in terms of ion permeation: AQP1, AQP6 and AtPIP2;1.

#### 3.2.1. AQP1 as a cGMP-Activated Cation Channel

After some initial controversial reports [15,87], Yool’s group demonstrated by means of different experimental approaches the fine-tuning mechanism of AQP1 as a nucleotide-activated ion channel that permeates cations—Na^+^ or K^+^— [16]. The hypothesis for cation permation through AQP1 requires cGMP-binding and a series of conformational changes in loop D for allowing cation permeation through the central pore [88].

AQP1 shows ion currents that are blunted by mercurial treatment when expressed in Xenopus oocytes and exposed to intracellular cGMP analogs [89]. Site mutagenesis experiments directed to the cGMP-binding region of AQP1 confirmed the sensitivity of AQP1 to cGMP [88,89,90]. Moreover, mutations in loop D interfered with ion channel activation but produced little effect on water channel activity [91]. In addition, MD simulations performed to study possible conformational changes after cGMP-binding predicted movements of loop D. As far as from now, no crystallographic evidence can support these observations [91]. It was also shown that phosphorylation of a carboxy-terminal tyrosine (Y253) enhances the availability of human AQP1 to be gated as an ion channel in response to cGMP, and this mechanism has been proposed as a master switch regulating the displacement of loop D [88,92].

Phosphorylation was also proposed to trigger the gating mechanism for ion transport through Big Brain AQP (BIB), an AQP from *Drosophila melanogaster* with very low water permeability. When expressed in oocytes, BIB is a monovalent cation channel reversibly activated by tyrosine kinase signaling without any appreciable water channel activity [93,94].

Which is the proposed pathway for cation permeation? As mentioned before, permeation of positive charged substances through the monomers of AQP1 is electrostatically restricted by the two concerted cation filters [81]. Differential effects of pharmacological agents on water and ion conductance were the first experimental evidence against cation conduction through the monomers of cGMP-activated AQP1 [95,96]. Since the AQP1 monomers were discarded as permeation pathways for the reported cGMP-induced cation conductance, the central pore became the new candidate for the ion permeation pathway in the subset of aquaporins with ion channel activity [88,97,98]. Molecular dynamics studies suggested that the occupancy of cations inside the hydrophobic central pore increases its size, enhancing ion permeability [91]. Site-directed mutagenesis studies were performed to confirm this hypothesis [88]. The central pore of human AQP1 is lined by four hydrophobic residues named VLLL (Val50, Leu54, Leu170 and Leu174) [88], located in transmembrane segments 2 and 5 (TM2 and TM5) [91]. When these hydrophobic residues are substituted by alanine, larger cations as tetraethylammonium (TEA^+^) can permeate AQP1 in response to cGMP [88]. In addition, differential inhibition of cation permeability was reported when a cysteine residue inside the central pore is replaced for a lysine [88]. Recent findings employing specific antagonists for blocking cation permeability but not the water permeability of cGMP-activated AQP1 have also reinforced the central pore hypothesis [92,99]. Single channel evidence in favor of the cGMP-gating of AQP1 was also reported by reconstitution into lipid bilayers. These experiments showed a large single channel conductance of approximately 150 pS [89] with apparently smaller sub-conducting states [96].

#### 3.2.2. AQP6 as an Intracellular Gated Anion Channel

AQP6—an AQP with low water permeability—was proposed to permeate anions in a pH-sensitive manner [100,101]. These findings imply a pH gating mechanism present in this atypical AQP, although the molecular details of the involved conformational changes are still unknown. Also, Hg^2+^ induces the appearance of anionic currents and the increase of water permeability of RnAQP6 expressed in *Xenopus laevis* oocytes [100,101,102]. Two cysteine residues responsible for Hg^2+^-activation were identified, and the double mutant C155A/C190A cancelled the Hg^2+^-activation. It is intriguing that the binding of Hg^2+^ to the corresponding sites (Cys189 in human AQP1 and Cys190 in rat AQP6) leads to inhibition of AQP1 but activation of AQP6. The authors suggest that Hg^2+^ appears to trap rat AQP6 in a conformational state that is permeable to water and ions [102]. This pharmacological activation of AQP6 with Hg^2+^ leaves the pore in the maximal open state. However, it must be emphasized that as far as from now there is no physiological evidence for promoting this open state of the channel. It was suggested that if AQP6 was confined in intracellular vesicles, then local pH might regulate its activity [102]. Activation of AQP6 by Hg^2+^ produces a less selective anion current than pH-activation—which allows cation permeation—and this might imply differences in both activation mechanisms. At the cytoplasmic mouth of the monomeric pore there is a positively charged residue (K72) that when mutated to a neutral charge generates an AQP6 with a non-selective current. These results suggest that this positive charge is crucial for AQP6 anion selectivity [100]. In the case of AQP6, cations can permeate through the monomers and Hg^2+^ can activate currents, therefore positive charges are not strictly repelled by NPA motifs as it was well established for AQP1 [82,83].

Although still speculative, the cation exclusion through the monomers of AQPs with conserved NPA motifs may not be a general feature. Another particular residue (T37) located opposite the Asparagines of the NPA motifs and not present in other mammalian AQPs, was reported to enhance NO_3_^−^ permeability and to participate in anion permeation [101]. All this evidence supports the idea that AQP6-monomers have a peculiar pathway for permeation [100,101,102]. Other unique residue accounting for the structural peculiarities of AQP6 is Asp60, located at the crossing point between helixes 2 and 5, a site that is strictly conserved as a Glycine in the rest of mammalians AQPs. Glycines located at this position mediate the surface interaction between TM2 and TM5 from adjacent monomers in other AQPs [19]. Substitution of Asn60 by Glycine converts AQP6 in a water permeable channel eliminating ion permeation [103]. Moreover, mutation of a related residue in the same region of AQP5 (L51R) induced anion permeability in the four individual pores and cancelled the water permeability [104]. The rigidity of the protein structure could also account for the water versus ion permeation. For maintaining the efficient and continuous passage of water molecules within a narrow open pathway of 3–4 Å and 20 Å-length, the helixes show a certain rigidity, and alterations of this rigidity may allow helix displacement, expanding the pore diameter and allowing anions translocation [83,104]. Single channel assays performed in AQP6 showed a unitary conductance of approximately 46 pS in standard physiological saline conditions [102].

#### 3.2.3. AtPIP2;1 Can Transport Ions

Up to now, limited evidence on ion transport through plant AQPs was available [105,106]. However, AtPIP2;1 (orthodox aquaporin) has recently been described as the first plant aquaporin reported to permeate cations when it is expressed in *Xenopus laevis* oocytes and yeast [107,108]. Results confirmed a non-selective current with similar regulatory mechanisms to those previously reported for its activity as a water channel [34], i.e., the current is increased at both low extracellular calcium concentrations and alkaline extracellular pH. Moreover, in AtPIP2;1 the ion current increases with a biphasic response by reduction of the external calcium concentration [107], which resembles the biphasic response reported on the water permeation of BvPIP2;1 [52]. These similarities between water permeability and ionic conductance (calcium and pH) led to the interpretation that both water and ions share the same permeation pathway, i.e., the monomeric pore. Evidence in favor of this hypothesis is supported by a naturally tryptophan substitution occurring in a PIP member (VvPIP 2;5 cv. C. sauvignon) that generated a non-functional water channel when expressed in *X. laevis* oocytes. Based on homology modeling performed with the structure of SoPIP2;1, it was proposed that this constitutive tryptophan substitution occurs in a well conserved Glycine position (W100) located in loop B, blocking the water passage through the monomer [109]. Then, based on this naturally occurring residue substitution, site mutagenesis performed in AtPIP2;1 (G103W) cancelled both the water permeability [110] and ion conductance [107] without affecting its trafficking capacity [110]. Although more studies are necessary to understand the cation permeation pathway of AtPIP2;1, this evidence favors the role of the monomer hypothesis for ion permeation [16,107], in contrast to the relevance given to the central pore in AQP1 [111]. Although anion permeation was not observed in AtPIP2;1, the cation permeation was reported to be regulated by extracellular Cl^−^ [107]. Interestingly, it was observed that AtPIP2;1-AtPIP1;2 heterotetramers blunt the ion transport but not the water permeability [107]. This evidence may suggest that heterotetrameric rearrangements can affect selectively permeation, as was previously shown for water permeation with tetramers from other plant species [53]. All this evidence reinforces the concept that certain AQPs have monomers that can expand their functional properties when interacting with the rest of the tetrameric components.

The existence of aquaporins that can also allow ion permeation requires modifying the present classification of this channel superfamily established into orthodox and non-orthodox AQPs. In recent years, some authors have defined these specific AQPs as “dual ion and water aquaporins” [16,111]. Interestingly, the controversial issue of ion-permeating AQPs had contributed to deep inside the required structural modifications that correlate with permeation events as well as introducing in the discussion more complex issues regarding the role of the central pore and putative permeation pathways.

During the last years, permeation of gases has been explored both in plant and animal AQPs—for a review see [11]. However, there is still no explicit evidence of conformational changes regulating the transport of gases through AQPs and there is very limited discussion about the putative permeation pathway [112]. Although some ion-permeating AQPs also evidenced gas permeation, the pathway for ions (central pore versus monomers) remains elusive.

Is there information in gas-permeable AQPs that can contribute to understand the role of conformational changes in permeation? Although experimental evidence indicates that AQP1 can permeate CO_2_, NO and NH_3_ [113,114,115], gas permeation through AQPs was questioned by theoretical and experimental approaches [115,116,117]. While experimental evidence using 4,4′-diisothiocyanato-stilbene-2,2´-disulfonic acid (DIDS) suggested that one half of CO_2_ permeation occurs through the central pore and the other half through the monomers [118], MD simulations predicted a preferential CO_2_ transport through the hydrophobic central pore of AQP1 over the monomers [119]. Other MD simulations also predicted that the preferential CO_2_ permeation through AQP1 over the lipid bilayer would occur depending on the membrane lipid composition [120].

CO_2_ and NH_3_ permeation events were also reported to occur through both AQP6 and the G60N-AQP6 mutant [115]. This mutant lacks anion-permeation capacity [103], suggesting that gas and ions are not sharing the permeation pathway. Recently, it was reported that AtPIP2;1 is also permeable to CO_2_, and that this permeation is blocked in the G103W-AtPIP2;1 mutant [107]. As water permeation is also blunted [107,110], CO_2_ translocation is potentially occurring through the monomers, as was proposed for cations.

### 3.3. AQPs That Sense Membrane Tension Changes: The Role of the Tetrameric Structure

From early studies, experimental results suggested that both animal and plant water channels could be regulated by osmolarity [121,122,123,124,125,126]. Indeed, it was proposed that AQPs act as osmosensors, transducing the osmolarity changes of the medium that surrounds the cells by means of a cooperative mechanism [127]. Subsequent evidence showed that intracellular volume or pressure can regulate the function of water channels, giving origin to the mechanical regulation hypothesis [128,129,130,131,132]. Then, the regulation of AQPs by a direct effect of membrane tension changes was tested for human AQP1 by mathematical modeling and simulation [38]. The results of this work indicate this mechanism can be reversible and support the osmosensor hypothesis [127,133] since data adjust to a positive cooperative mechanism that could involve the four subunits of the tetramer [38]. Almost simultaneously, experimental evidence supporting the regulation of VvTIP2;1 (grape) by membrane tension changes was reported [132].

Experimental demonstration for the tension-mediated regulation in animal and plant AQPs was reported by simultaneous measurements of mechanical coefficients and water transport rates in proteoliposomes with RnAQP4 (rat) [134] and Xenopus oocytes expressing BvTIP1;2 (beet) [135]. In both cases, the water permeability coefficient decreases in a nonlinear fashion with increments of membrane elasticity, as was predicted for AQP1 [38]. These results show that AQPs respond to membrane tension changes in the opposite way that mechanosensitive (MS) ion channels do. As widely reported on revisions, MS ion channels activate (open) with increasing tension [136,137,138,139]. On the contrary, MS AQPs (AQP4, AQP1, BvTIP1;2, VvTIP2;1) decrease their water transport rate when membrane tension raises, evidencing a closure event of the water pathway [38,132,134,135]. However, it cannot be generalized since MD simulations performed with the structure of AQY1 from the yeast *Pichia pastoris* predict that this AQP would open when increasing membrane curvature [64].

In analogy with the study of ion channels, the transport capacity of AQPs can be evidenced in a plot of water flux versus osmotic gradient (J_w_-Δ_osm_). For mechanosensitive AQPs such as human AQP1 and BvTIP1;2, the J_w_-Δ_osm_ plots show deviations from linearity with gradients higher than 100 mosmol·Kg_w_^−1^ [38,135]. This deviation shows that P*_f_* is not a constant value. In fact, since the measured J_w_ is lower than expected, then there is a decrease of P*_f_*. This was initially supposed to be originated by two alternative hypotheses: (1) the water passage through the water pore could have a limiting step, or (2) the result could be consequence of a gating mechanism mediated by membrane tension [126]. Updated evidence suggests that these two hypotheses could be part of the same mechanism. In the next paragraphs we review the facts that support this proposal.

As was exposed in Section 2.2, molecular basis of water permeation indicates that P*_f_* values are dependent on the number of H-bonds that water molecules form with pore-lining residues [54]. In addition, results obtained with rat AQP4 reconstituted into proteoliposomes formed by phospholipids of different length showed that p*_f_* decreases with increasing the bilayer thickness, evidencing a hydrophobic mismatch effect [140]. Moreover, this p*_f_* decrease concurs with a geometrical change of the pore entrance at the cytoplasmic side. The pore radius increases changing the angle of the conical shape of the cytoplasmic entrance [140], where H-bond forming Glycine residues are located [56]. Similar results were predicted by simulations performed by combining analytical modeling with finite-element calculations in artificial nanopores. These simulations show that the conical entrance of the pore optimizes its unitary water permeability and that the best angles are similar to those observed in AQPs [141]. This evidence suggests that increasing the diameter of the pore entrance can affect the optimal distance for H-bonds between water molecules and the pore-lining residues.

Revisions on MS ion channels hypothesized that mechanical regulation would be a very ancient trait [137,138]. According to the pool of evidence, a MS ion channel must have a helix (a sensor) directly coupled to a pore-lining helix to be gated by the lateral tension of the membrane [139]. Usually, this coupling is mediated by Glycine residues. Thus, it was proposed that the GxxxG motif, which is characteristic of transmembrane segments [142], is also responsible for mediating mechanotransduction [143]. These features characterize the gating mechanism of MS ion channels, considering that the pore is formed by transmembrane segments from different subunits that change their position in the membrane [136]. Unlike to MS ion channels, each monomeric subunit conforms a water pathway in AQPs, and the transmembrane segments lining the pore constitute a rigid structure. This feature is one of the reasons why so many structures of AQPs were resolved with very high resolution (~2 Å) [11]. Moreover, the key structural feature of AQPs are the long loops B and E that immerse deep inside the lipid bilayer, setting two short α-helixes, H-bond forming residues, the two NPA motifs and the selectivity filter in the core of the membrane [11].

Besides the differences between AQPs and ion channels, the GxxxG sequence appears several times in the primary structure of AQPs (6 times in human AQP1). Intriguingly, this sequence is present not only in TM segments but also in loop B, just before the first NPA, a key region for water transport [135]. In addition, the Glycine and Histidine residues that form H-bonds with water molecules at the cytoplasmic entrance before the single-file region constitute the GxxxG sequence in AQPs [54,56].

This evidence gives a picture of the stepwise movement of water inside the pore. However, beyond the monomeric scale, other reports show interactions between monomers and that water transport rates can be modified by heterotetrameric conformation. The tetrameric structure of AQPs is stabilized by interacting forces between TM1 and TM2 from one monomer with TM4 and TM5 from the adjacent monomer [19,144]. These interactions are mediated by Glycine residues. In addition, the intracellular loop D contributes to tetramer stabilization [145].

At the other side of the membrane, the structural differences of extracellular loops from different types of AQPs are related to differential interactions with the lipid bilayer. In the orthodox aquaporin Z (AQPZ) from *E. coli* [146], the extracellular loop C is shorter than in the aquaglyceroporin GLpF [147], which possesses a more pronounced secondary structure. Thus, the height of the protrusions registered by atomic force microscopy (AFM) is shorter in AQPZ than in GLpF. High-speed atomic force microscopy (HS-AFM) records demonstrated that while lateral stiffness of loop C from GLpF is slightly dependent on the plane of the membrane (X-Y axis), the lateral stiffness of loop C from AQPZ is asymmetric, being less stiff in the direction connecting helixes 3 and 4 (TM3 and TM4) than in the perpendicular direction [147].

Evidence from different plant species (maize, rice, strawberry, red beet) show that PIP1 and PIP2 isoforms can form heterotetramers with different stoichiometry [39,53,148,149,150,151]. Both loops A and D seem to be important for tetramer stabilization and modulation of the water transport rate [148,149]. In strawberry and red beet, PIP1-PIP2 heterotetramers show different water permeability values according to the stoichiometric combination of each type of monomer [39,53,79], demonstrating that the different water transport capacity exhibited by PIP1-PIP2 heterotetramers is consequence of the environmental features faced by a monomer, which are determined by its neighbors. In accordance to this, recent MD simulations predict that TM-segment interactions in PIP1-PIP2 heterotetramers from maize modulates the water permeability of PIP1 monomers [152].

Since there is a hydrophobic coupling between protein function and bilayer material properties, Lundbaek and coworkers stated that “the bilayer becomes an allosteric regulator of membrane protein function” [153], as was previously cited [134]. Molecular dynamics simulations predicted that the interaction of sheep AQP0 (PDB 2B6O) with the first shell of lipids are mediated by H-bonds that are not restricted to one phospholipid molecule but rather to all the lipids (7–9 lipids) forming the annular shell around the tetramer [154]. Instead of being a fixed shell, as occur with some ion channels, the annular lipids around AQPs are predicted to be interchanged with the bulk lipids of the membrane, and that this pattern would be conserved in all AQPs [155].

**Figure 3:**
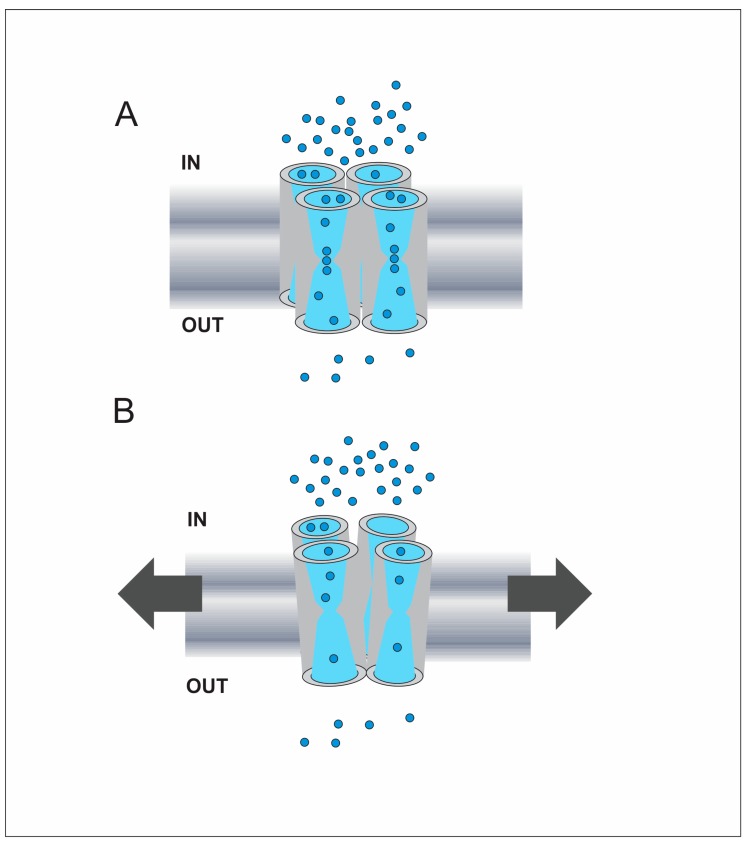
Schematic representation of AQP regulation mediated by membrane tension increments. (**A**) Illustration of a tetramer in a membrane subjected to basal tension. The applied osmotic gradient is represented by the different amount of water molecules (light blue circles) between extracellular and intracellular compartments (OUT and IN, respectively). The water pathway is schematically represented in each monomer. Since the membrane is not subjected to extra tension at the beginning of the osmotic response, the four monomers are not subjected to extra strain and the water permeability is high. (**B**) The development of the osmotic response produces increments of cell volume and pressure, and hence the increase of membrane tension [38,135], represented by arrows. This could produce slight distortions on the tetramer as well as slight distortions on each monomer. In mechanosensitive aquaporins we propose that these changes affect the distance between water molecules and the pore-lining residues that participate in H-bonds, e.g., at the cytoplasmic entrance to the single-file region [140]. Consequently, the probability for a water molecule to form H-bonds with pore-lining residues would be lower, producing a decrease of the unitary water permeability [54]. These events that occur in each monomer could be affected by the interactions with its neighbors, what could produce a cooperative effect for P*_f_* decrease [38]. This scheme is inspired on the cooperative mechanism proposed by Hill et al. and used to formulate the osmosensor hypothesis [127,133].

Based on the evidence reviewed in this section, a possible mechanism for membrane tension regulation can be proposed (Figure 3). Membrane tension increments can be translated to the tetramer by direct interaction between the protein and the lipids [147,154,155]. Then, the increase of membrane tension could modify the balance of the hydrophobic mismatch between the protein and the lipids [140], which could produce slight distortions of the monomers. Next, this could increase (or at least modify) the distance needed for water molecules to form H-bonds with pore-lining residues, as e.g., at the cytoplasmic entrance before the single-file region [140], where key Glycine and Histidine residues are located [56,58]. This would result in a decrease of the probability for water molecules to form H-bonds with the protein. Since the water permeability values correlates with the number of these H-bonds [54], then the monomeric water permeability would decrease. In addition, this could be affected by the interactions among monomers [19,144,145], which could produce a positive cooperative effect on P*_f_* decrease [38].

### 3.4. Beyond the Tetrameric Structure

Although the tetrameric structure of almost all AQPs determines their function as water channels, some AQPs conform supramolecular assemblies allowing membrane-membrane interactions. Some examples of interactions between cells (junctions) and within the same cell (arrays) where observed [156]. Thus, the term “adhennel” has been coined for membrane proteins that function both as a cell-adhesion molecule and a membrane water channel [157]. The structures of supramolecular assemblies and the mechanisms that regulate assembly formation have been extensively investigated in AQP0 and AQP4 [66,67,157], observing some similarities between them [41]. Both AQP0 and AQP4 conform orthogonal arrays in vivo (native membranes) [158,159] and were found in membrane junctions that are probably constituted by these arrays [157,160]. Also, the existence of long and short forms has been reported in both proteins. In AQP0 these forms are produced because of proteolytic cleavage [161], and in AQP4 because of the expression of two splicing variants (M1 and M23) [162]. The shorter protein species are most likely to conform orthogonal arrays, particularly in the case of AQP4 [163,164]. Regarding functional aspects of these isoforms, expression of the truncated form of AQP0 in *Xenopus laevis* oocytes suggests that cleavage does not affect the passage of water [165]. Also, both isoforms of AQP4 were observed to have the same water permeability [115]. However, structural evidence indicates that junction formation closes the water channel of AQP0 [66,67] and obstructs the extracellular channel entrance in AQP4 [157].

#### 3.4.1. AQP0

It is well known that the proteolitic cleavage in the cytoplasmatic side is a necessary step to the junction formation. After cleavage, structural changes occur, followed by the alignment of AQP0-tetramers from two adjacent cells. Thus, tetramers face each other from the extracellular side and the junction is stabilized by interactions between residues of extracellular loops A and C [66]. At the center of the tetramer a rosette-like structure is formed, mainly mediated by P38 [67]. Also, R33 is critical in the junction formation. Substitution of this Arginine by a Cysteine diminishes cell-adhesion properties in vitro [166] and causes congenital cataracts in humans [167]. On the other side, experiments in *Xenopus laevis* oocytes demonstrated that proteolytic cleavage does not affect the water permeability of AQP0 [165]. Structural studies suggest that junctional AQP0 is in a closed state [66] and molecular dynamics simulations indicate the junction does not affect P*_f_* [168]; however, functional evidence on transport properties of junctional AQP0 is still lacking. An unusually narrow water permeation pathway is observed in AQP0 [168,169]. The monomeric pore contains only seven water molecules [67,168] instead of the eight or nine present in other AQPs [170]. A particular Tyrosine residue near the Ar/R site (Y23 or Y24) [156,171,172] interrupts the H-bonds continuity at the center of the water channel [168,171]. An additional constriction region formed by residue Y149 in AQP0 [171] interrupts the H-bonds continuity, which seems to be necessary to allow water conductivity [54,69] and affects the electrostatic profile of the pore [84]. However, a closed state of AQP0 is thought to be achieved after the junction is formed. In this closed state, the number of water molecules in each monomer was shown to be reduced to only three, with distances too far apart to form H-bonding contacts [67,156]. Two residues were proposed to be crucial in this H-bonding interruption, a Methionine (M176) at the Ar/R selective filter and a Tyrosine (Y124) at the cytoplasmatic side of the pore [67]. Therefore, the access of water to the single-file region is restricted from both the cytoplasmic and extracellular sides of the channel. Molecular dynamics simulations indicate that both the low water permeability in the junctional and non-junctional AQP0 is a consequence of the combination of both a static barrier and dynamic gating motions [168]. These conformational changes in the closed state of the junctional AQP0 are not observed in the cleavage protein, suggesting the idea that interaction during the junction formation and not the cleavage per se produces this closure [67,172]. A reorientation of the Y149 [60] was proposed to occur after the protonation of H66 [66]. Recently this histidine was reported to be crucial in pH sensitivity of AQP0 [68].

#### 3.4.2. AQP4

AQP4 is usually observed in two isoforms (M1 and M23). Although M1-AQP4 exists mainly as individual tetramers [173], the formation of square arrays is observed only in presence of the shorter isoform, M23-AQP4 [164]. The formation of these arrays is supposed to not affect per se the water osmotic permeability of AQP4 because similar P*_f_* values are observed when the two isoforms are expressed in *Xenopus laevis* oocytes. However, the possibility of differences in the permeation capacity has been discussed as the M23 isoform can permeate CO_2_ but M1 has impaired gas conduction [115]. Interaction of tetramers in a junction arrangement requires an organized array [156]. As reported in AQP0, interactions of unique Prolines in the extracellular domain of AQP4 were proposed to stabilize the junction formation [56,157]. These Prolines were not observed in other mammalian AQPs [156]. However, in AQP4 one tetramer is proposed to interact with four other tetramers in the adjoining membrane, partially blocking the water-conducting channel [41,156]. Then, on one hand, AQP4-junctions must be formed only in the case of orthogonal arrays, and on the other hand, a water permeation junction must constitute a resistance for water permeability across the two membranes because of the partial blockage of the water pores.

Although AQP4 may form junctions, there are controversial results regarding the cell adhesive properties in vivo. In glial cells, junctional membrane zones were observed and AQP4 was detected by gold labeling [174]. Also, increased cell-adhesion properties of AQP4-expressing cells were detected [157]. However, when different cell lines were evaluated, AQP4 did not promote cell adhesion. Moreover, binding of the AQP4 extracellular domain to a peptide, proposed to promote tetramer interaction, also failed [175]. Different permeation molecules and cell-adhesion properties of junction formation water channels—such as AQP0 and AQP4—remains elusive. Therefore, further explorations of these properties are needed. Indeed, new candidates may be identified, as for example SoPIP2;1, which has also been proposed to form double-layered arrangements [41,176].

## 4. Conclusions and Perspectives

Recent research in the field of AQPs has increased our knowledge of their regulation mechanisms. Nowadays, we have information that embraces functional changes with molecular details. New evidence reinforces the concept that certain AQPs can expand their membrane properties when their functional monomers interact with each other.

The impact of aquaporins on water membrane permeability adjustments as a response to physiological processes must be taken into consideration when analyzing physiology and pathophysiology [27,28,29,30,31]. Solute-permeable aquaporins are also unexpectedly emerging as critical selective transporters with physiological implications [9,10,11,12]. However, ion-permeable aquaporins not only represent a challenge in the understanding of their physiological role in biological membranes as ion transporters but also because they promote the study of the molecular basis of ion permeation in these proteins as a whole. This is of particular interest in AQPs that are well known for being highly permeable water channels [89,90,91,92,107]. Also, this subject can introduce some new light in the role of the central pore. Furthermore, exploring the impact of conformational changes in permeation with new approaches could be relevant for the study of specific ion-permeable aquaporins. Also, new unexplored questions could be raised.

When considering the functional monomer and its capacity to transport water, evidence shows that water permeability in AQPs is governed at the molecular level by the ability of water molecules to form H-bonds with pore-lining residues and, (if applies) by the restriction of the water pathway by certain mechanisms (e.g., pinching or capping). However, in certain AQPs, this capacity is affected by the interactions between monomers, as demonstrated by heterooligomerization or response to membrane tension. Evidence also confirms that in certain AQPs the tetrameric conformation is modulated by interactions with the lipid bilayer or even with other tetramers—or other proteins—in supramolecular arrangements. Thus, from the monomer to the tetramer, different levels of regulation can operate and in consequence increase the output response.

Two recent revisions explore simultaneously animal and plant aquaporins with the aim to integrate the knowledge of aquaporins inside a unique mainframe [11,177]. One unresolved issue in this approach of integration is to pursue (if plausible) for a consensual nomenclature that could improve our approach to seek information that is relevant. In previous work we proposed, among others, to find a classification based on the evolutionary framework that could also help to improve our capacity to predict permeation/selectivity and structure-function features [178,179]. Between the described animal membrane intrinsic proteins (MIPs) and the plant subfamilies there is possibility to restrict to four ancestral subfamilies: (A) AQP1-like and PIP, (B) AQP8-like and tonoplast intrinsic proteins (TIP), (C) AQP3-like and nodulin26-like intrinsic proteins (NIP), and (D) AQP11-like and the SIP subfamilies [178]. If we consider the abovementioned framework, we can show that almost all ion-permeable AQPs described in this review belong to subfamily (A). Also, growing evidence of lactic acid transport can be found in AQPs belonging to subfamily (C) [83], which includes the anion channel soybean NOD26 [105]. No evidence of ion transport in the subfamily (B) or (D) is available. On the other hand, mechanosensitive aquaporins are as far as from now restricted to subfamily (A) and (B). The aquaporins are ancestral channels as are some of their traits and this must be taken into consideration. Thus, this framework or an improved version might contribute to better search for connections.

Finally, we found the breakthrough in the field since the AQPs were discovered encouraging, as well as how much remains to be explored.
